# Global and Regional Vigilance: Are There Two Types of Local Sleep?

**DOI:** 10.1002/brb3.71362

**Published:** 2026-03-31

**Authors:** Tom Deboer

**Affiliations:** ^1^ Laboratory For Neurophysiology, Department of Cell and Chemical Biology Leiden University Medical Center Leiden the Netherlands

**Keywords:** local sleep, parasomnias, slow‐waves

## Abstract

**Purpose:**

Since the discovery of uni‐hemispheric sleep in dolphins we have accepted that sleep does not need to be a global cortical state. The term “local sleep” has been introduced for this phenomenon.

**Finding:**

However, the term seems to be used for two distinct types of local sleep. The first type, which is seen in dolphins, is the one where two different vigilance states are found simultaneously on the cortex like in non‐rapid eye‐movement (NREM) sleep parasomnias in humans. In the second type, only one vigilance state is observed, but the activity of local neurons is changed by changing their workload during previous waking. There are not only functional, but probably also mechanistic differences between the two types of local sleep, where the first originates from deeper brain areas, and the second is induced locally on the cortex.

**Conclusion:**

Recognizing this distinction helps to ask clearer defined questions when investigating local sleep.

## Graphical abstract

The term local sleep as it is used in the literature is confusing and probably consists of two types of brain states. The first one originates from increased activity in certain cortical areas during waking, resulting in increased activity in the slow‐waves range in that same area during subsequent NREM sleep. The second one consists of local states in a global state (i.e., waking during NREM sleep), which are likely to originate from an imbalance in the activity of the ascending reticular arousal system.

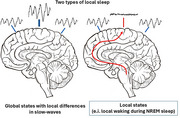

## 1 Introduction

There is a phenomenon that has entered the sleep field approximately 30 years ago, which is the concept of “local sleep.” It was inspired by the finding that dolphins can sleep with only one hemisphere at the time, demonstrating that a vigilance state does not need to be expressed globally over the entire cortex. On the basis of this sleep, researchers started to develop ideas about local differences between cortical areas and demonstrated that the human brain can also show local differences in cortical activity. However, there is some unclarity about what local sleep is, and it seems there may actually be two types of phenomena, possibly with two different mechanisms, which are both called local sleep. The two types of local sleep may be connected to two main sleep research areas in which the sleep field is divided.

The first area investigates the interaction between brain areas and how they together decide whether the brain is asleep or awake, and when asleep how the two main sleep states, non‐rapid eye‐movement (NREM) and REM sleep, are alternating in a regular fashion. We think we understand most of the interactions occurring between different brain areas involved and how they regulate the vigilance states in such a way that they occur in a stable and predictable fashion (Saper et al. 2005). The second area mainly investigates the way the brain makes the decision on when to fall asleep. From that area we know that sleep deprivation increases the tendency to go to sleep and that a sleep debt in one way or another needs to be recovered or compensated. Here, the circadian clock is taking part in the decision to fall asleep and wake up (Borbely et al. 2016). We do not exactly know how the two main fields can be brought together (Deboer 2025), but that may just be a matter of time.

This perspective summarizes the development of the idea of local sleep from dolphins to humans and from differences in vigilance states between two brain hemispheres down to the question whether single neurons on the cortex of a healthy brain always need to participate in the neuronal group activity. Do neurons need to follow the global state, or are neurons allowed to desert the army and run their own rhythm, showing “local sleep” in a single neuron? Eventually we end up with the question what we mean when we talk and discuss about local sleep. Do we mean the same or is there maybe more than one type of local sleep?

## 2 How We Think the Brain Changes States: The Flip‐Flop Model

**FIGURE 1:**
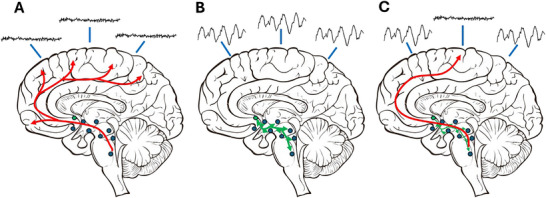
(A) A schematic drawing showing the key elements of the ascending arousal system. Different areas in the thalamus, pons, and laterodorsal tegmental nucleus, together called the ascending reticular arousal system or ARAS (blue dots), release acetylcholine and monoamines, which facilitates thalamocortical transmission and helps to induce and maintain a cortical wakefulness state (as represented by schematic EEG traces of waking EEG). (B) A schematic drawing indicating how the ventrolateral preoptic area (green dot) releases GABA and galanin to inhibit the different areas constituting the ARAS, inducing a global state of sleep (as represented by schematic EEG traces of NREM sleep EEG). (C) Proposed mechanism how reduced inhibition by the VLPO or increased activity of part of the ARAS can induce a local waking state within a globally sleeping cortex, as seen in human NREM sleep parasomnias.

Most brain areas involved in switching between vigilance states can be found in the hypothalamus and the pons (Figure 1) (Saper et al. 2005; Luppi et al. 2011). From here, two ascending pathways promote and maintain waking (Figure 1A). One pathway runs from the pons to the thalamus and activates thalamic relay neurons. It mainly consists of acetylcholine‐producing neurons in the pedunculopontine and laterodorsal tegmental nucleus (Hallanger et al. 1987). They are active during both waking and REM sleep, but less active during NREM sleep (McCormick 1989). The other pathway originates from different groups of monoaminergic neurons, including the serotonergic raphe nucleus, the dopaminergic ventral periaqueductal gray matter, the histaminergic tuberomammillary neurons, and noradrenergic neurons in the locus coeruleus. These project to the lateral hypothalamus, the basal forebrain, and large parts of the cortex (Jones 2003). These pathways are most active during waking, less active during NREM sleep, and silent during REM sleep (Aston‐Jones and Bloom 1981; Steininger et al. 1999;; Oikonomou et al. 2019). The consensus is that these two pathways, together called the ascending reticular arousal system (ARAS), stimulate waking.

On the opposite side stands the ventrolateral preoptic area (VLPO), which is thought to induce and maintain NREM sleep (Figure 1B). The VLPO is mainly active during sleep and has GABA‐ergic outputs to the acetylcholine producing cells and the different monoaminergic cell groups involved in wake maintenance (Gaus et al. 2002 Sherin et al. 1998;; Szymusiak et al. 1998).

This reciprocal inhibition in sleep‐wake regulation was found to resemble a self‐reinforcing loop, which in electrical engineering is called a “flip‐flop” switch, and therefore this term was coined for this sleep‐wake regulatory system (Saper et al. 2005), and is in this way used to describe this network. With a flip‐flop switch, transitional states are mainly avoided, which explains why transitions between waking and sleep are generally experienced as abrupt. However, a flip‐flop switch shows continuous switching between states when not controlled. Orexin is released by specific neurons in the lateral hypothalamus, which are mainly active during waking. Orexin levels increase in the course of the main waking period and during sleep deprivation (Deboer et al. 2004 Zeitzer et al. 2003) and is thought to stabilize the sleep‐wake flip‐flop switch (Saper et al. 2005)

In this anatomical network, regulating sleep and waking initiation and termination, the cortex is not an active player. It merely receives waking or REM sleep activity inducing input from the ARAS, and when this activity is inhibited by the VLPO, the cortex is, more or less, left on its own and switches to a mode of activity that we associate with NREM sleep.

## 3 NREM Sleep Homeostasis and Circadian Sleep Regulation

In the other area of sleep research we enter a field where the cortex does seems to have some role in sleep‐wake regulation. Here, the two‐process model of sleep regulation is one of the most influential models that frames the view through which sleep is analyzed (Borbely et al. 2016;; Borbely 1982). The slow waves in the NREM sleep EEG reflect the depth of sleep and are an indicator of sleep debt (see also the text box). The longer someone has been awake, the more slow waves are visible in the EEG during NREM sleep, and the longer someone has been in NREM sleep, the lower the activity of the slow waves will be. They are supposed to reflect a homeostatic process, called process S, that keeps track of the duration of prior wakefulness as such that the level of this process is reflected in the amplitude and the density of EEG slow waves, which together determine the level of slow‐wave activity in the EEG power density spectrum during NREM sleep. This process is thought to be one of the two processes that determines the timing of switching between sleep and waking. When, during waking, process S reaches an upper threshold, the brain falls asleep. When in the course of sleep it reaches a lower threshold, the brain wakes up. These two thresholds are not static and constant, but increase and decrease in a daily rhythm, regulated by the central circadian pacemaker, residing in the suprachiasmatic nucleus of the hypothalamus. How these processes are linked to the anatomical centers that eventually are responsible for the initiation of sleep and waking has been reviewed elsewhere (Saper et al. 2005; Deboer 2020).

For this model to work, and to determine the timing and amount of sleep and waking, the changes in cortical slow‐wave activity need to be, more or less, a global process, taking place at the same time in the same magnitude over the entire cortex. This is not completely the case (Huber et al. 2000;; Palchykova et al. 2002; Rusterholz and Achermann 2011), but the differences across different parts of the cortex are also not that large that it is impossible to still consider it as a global process.

## 4 From Global to Local Thinking

In the models mentioned above, the cortex is either a passive bystander, or it only works as a global representative of homeostatic sleep pressure. In the flip‐flop model, the cortex is influenced in its activity, but not actively participating in the decision‐making when it comes to transiting between vigilance states. In the two process model, the slow‐wave activity produced by the cortex as a whole is considered to reflect the level of process S, and the crossing of this process with the two thresholds for waking and sleep determines the timing of transitioning between these states.

Yet, we know that vigilance states and slow waves are not always the same all over the cortex. This started off with observations in dolphins that were shown to exhibit deep NREM sleep only in one hemisphere (Mukhametov et al. 1977). Experiments with selective sleep deprivation of one hemisphere in dolphins even resulted in a uni‐hemispheric rebound in NREM sleep (Oleksenko et al. 1992). More recent recordings in other aquatic mammals revealed that the northern fur seal also displays uni‐hemispheric sleep only when forced to stay in the water (Lapierre et al. 2007; Lyamin et al. 2008) and that this is associated with bilateral differences in acetylcholine release to the cortex (Lapierre et al. 2007), but no bilateral differences in the release of histamine, norepinephrine, and serotonin (Lyamin et al. 2016). Recent work in humans investigating the “first‐night effect” in the sleep laboratory in humans showed that one hemisphere sleeps less deep (shows less slow waves in NREM sleep) during the first night in the sleep laboratory (Tamaki et al. 2016), suggesting increased vigilance in one hemisphere in potentially dangerous situations. The data show that sleep, and sleep homeostasis, are not necessary global processes, running simultaneously across the entire brain. The discovery of unilateral sleep in dolphins was the basis of the idea that use‐dependent local cortical mechanism may underlie the sleep deprivation induced changes in EEG slow waves during NREM sleep (Benington and Heller 1995; Krueger et al. 1999).

This idea was first tested in pioneering work in the group of Alexander Borbely who showed that local activation of a particular cortical area during waking resulted in EEG changes recorded from the same cortical area during subsequent sleep (Kattler et al. 1994). They applied a vibratory stimulus to the hand, to activate the contralateral somatosensory cortex. The effects on the subsequently recorded sleep EEG was most prominent in the slow‐wave range of the EEG, and was restricted to the derivation that corresponded with the same cortical area. This was followed by research stimulating the barrel cortex via the vibrissae (Vyazovskiy et al. 2004), and stimulating the motor cortex with running wheel activity (Vyazovskiy et al. 2006) in mice, and by making use of the paw preference in reaching behavior in rats (Vyazovskiy and Tobler 2008). In another experiment, in humans, a motor learning task was applied, which involved increased activity in a known restricted part of the cortex (Huber et al. 2004). The experiment confirmed, in humans, that the activity of local cortical slow waves in the NREM sleep EEG can be increased by enhancing the workload of that particular part of the cortex during previous waking. The opposite was true when depriving the cortex of input by restraining the corresponding limb (Huber et al. 2006). A prominent hypothesis explaining the observed phenomena is the synaptic homeostasis hypothesis (Tononi and Cirelli 2006), which proposes that with increased use of the cortex during waking, more connections are made between these cortical neurons leading to increased synchronizations, and therefore increased SWA, during subsequent sleep. This is thought to occur globally on the cortex, but can differ locally as it is use dependent. The data indicate that the occurrence of slow waves and their homeostatic changes are a local cortical phenomenon that can be changed by manipulating the local cortical workload during prior waking.

## 5 Local States in a Global State

This effect of local manipulation of cortical activity is, however, different from the situation found in aquatic mammals, where two distinct states are simultaneously occurring on the cortex. There are a few conditions known, even in humans, where a vigilance state on a local‐cortical level can be observed opposite to the global vigilance state that is recorded from the rest of the cortex. One occurs in a situation of increased sleep pressure where local sleep is observed during global wakefulness. The other is during sleep walking or somnambulance (but think also about bruxism and other NREM sleep phenomena), where a sleeping person is walking and behaving and where it is shown that the cortex is probably, locally, awake within the global state of NREM sleep.

In the first condition, after prolonged wakefulness, the EEG during waking shows a gradual increase in slower waves (below 8 Hz) and this increase is a function of the time spent awake (Cajochen et al. 1995; Cajochen et al. 2002; Strijkstra et al. 2003), putatively reflecting the effect of a sleep homeostatic process, similar to slow waves in the NREM sleep EEG (Borbely et al. 2016). Based on electrophysiological data in monkeys, it has been suggested that the slowing of the waking EEG is associated with local sleep states during waking (Nobili et al. 2012). It was shown, in sleep deprived monkeys performing a visual discrimination task, that neurons in the visual cortex show a sleep‐like firing pattern, while at the same time neurons in the striatal cortex showed a wake‐like firing pattern (Pigarev et al. 1997). The episodes of sleep‐like firing characteristics, locally in the visual cortex, became more frequent in animals that seemed to experience more sleep pressure. The data suggested that the initiation of sleep onset at the cortical level is not as global as assumed by the flip‐flop model, but may occur at different time points, depending on the cortical area. Similar data were obtained in rats, where Vyazovskiy et al. (2011) showed that when sleep pressure is increased, in the awake animals, small populations of neurons in different cortical areas may show sudden off periods (see text box), which are usually associated with NREM sleep, whereas globally no further switching to off periods or NREM sleep were observed. In the same experiment, it was shown that the timing of the local cortical slow waves correlated with failures on a reaching task (Vyazovskiy et al. 2011). The number of local off periods increased with the duration of wakefulness, suggesting that the off states are associated with a local increase in sleep pressure, but lasted for only a couple of seconds.

In the second condition, sleep and wakefulness activity can be simultaneously observed in different parts of the cortex, but these are more stable and the cortical areas are larger. Particularly, the group of Lino Nobili has shown with EEG recordings in pharmacoresistant focal epilepsy patients that not the whole cortex is always in a sleep mode at the same time (Nobili et al. 2012). These patients had no known sleep disturbances and underwent intracerebral EEG investigations before surgical interventions for their epilepsy. It was observed that sleep was characterized by both wake‐like and sleep‐like EEG patterns in different cortical areas. Mainly it was found that local activation, characterized by faster frequencies in the EEG occurred synchronously with patterns of deep‐sleep EEG in the dorsolateral prefrontal cortex (Nobili et al. 2011). The number of local activations increased in the course of the night and increased toward the end of each NREM sleep episode. The authors suggested that the coexistence of the two states could be associated with the occurrence of certain motor phenomena observed in NREM sleep parasomnias.

These conditions mentioned above are all mixes of NREM sleep with waking. One may be able to also include REM sleep in this, although less is known about local REM sleep phenomena. For instance, slow waves have been recorded during REM sleep in superficial layers of the primary cortex (Funk et al. 2016), and transition from NREM sleep to REM sleep appear to occur heterogeneously across cortical regions (Durán et al. 2018; Emrick et al. 2016; Serantes et al. 2025). Also lucid dreaming (Tzioridou et al. 2025) may be a phenomenon caused by local wakefulness within REM sleep.

## 6 Two Types of Local Sleep?

**FIGURE 2:**
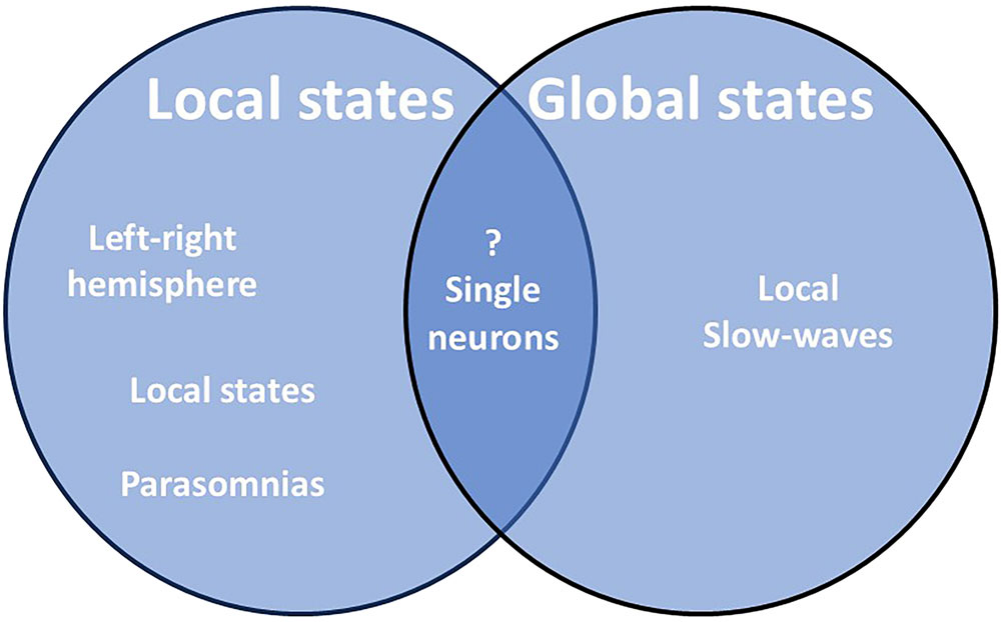
Division of different types of local sleep states depending on their putative mechanism of regulation. The local states are states of vigilance where the cortex is not globally in one single state, but displays at least two states in different cortical areas. These states are probably induced by local differences in neurotransmitter release from deeper brain areas as part of the ascending reticular arousal system. The global states show one single state across the entire cortex with local deviations in the level of slow‐waves, induced by differences in their amount of labor/firing shown during previous wakefulness. These are therefore differences induced by local differences in activity during previous wakefulness.

Both local manipulation of EEG slow waves and local states within global states are presented in the literature as examples of local sleep. But it seems that what we call local sleep is likely to exist out of two types of local sleep (Figure 2). One with clear separation of vigilance states on the cortex, the other with a single global vigilance state for the cortex, which is in general NREM sleep, but with local differences in the intensity of the state.

The first one is the one where the idea of local sleep originated. The cortex is in different states depending on the location on the cortex. This is clear in the dolphins and other aquatic mammals where one hemisphere is awake and the other is in NREM sleep (Mukhametov et al. 1977; Lyamin et al. 2008). Something similar, but with a less clear distinction of the cortical surface areas is occurring in the human brain putatively related to parasomnia (Figure 1C) (Nobili et al. 2012). Eventually this can even be observed during waking in smaller cortical areas under increased sleep pressure where it is associated with lapses in performance (Vyazovskiy et al. 2011). Finally, also during waking under increased sleep pressure, this can be observed in single neurons not following the global behavior, displaying a firing mode associated with sleep (Pigarev et al. 1997).

The second type is, until now, mainly observed in NREM sleep and is induced by intensifying the previous waking experience for a certain sensory input or task. This can be only sensory (Kattler et al. 1994) or a combined sensory and learning task (Huber et al. 2004) of which it is known that it locally increases cortical activity. During subsequent NREM sleep, a local increase in SWA is then observed. The difference with the first type is that the global state of the brain remains intact and that the local differences are mainly found in the occurrence and amplitude of particular waves, eventually showing local changes in EEG power density in particular frequencies, usually below 5 Hz.

Extending this idea, it may be that the two types of local sleep are induced by two different mechanisms. The first type may be induced by subcortical regions related to the ARAS. This idea is supported by findings in aquatic mammals’ uni‐hemispheric sleep characterized by differences in release of acetylcholine in the cortex of the different hemispheres, where in the awake hemisphere acetylcholine release is increased (Lapierre et al. 2007). This would mean that the cortical regional differences in vigilances states observed in other species, including humans, may also be caused by regional differences in the release of acetylcholine, or possibly one of the monoamines released by ARAS, normally involved in the induction and maintenance of cortical waking. The second type is a local diversion of global activity and seems to be mainly induced by differences in the level of activity between cortical areas during previous waking. This type of local sleep is not characterized by different vigilances states on the cortex, but by local differences in EEG activity within the same state, probably through changes in local connectivity or differences in local release of slow‐wave inducing substances, like adenosine, by the previously intensified waking activity (Reichert et al. 2022).

The situation of the single neurons or a small group of neurons showing sleep like behavior in a waking cortex is less clear, because they do not last long and are not detectable by EEG. They may be part of a transition between states, instead of being true local states as local states generally last longer. Alternatively, they may reflect differences in timing of recruitment of neurons to sleeping networks when falling asleep. However, they also may hold the middle ground between the two types being induced by local synchronization between neurons and simultaneously being “allowed” to occur through local differences in the input from the ARAS.

## 7 Conclusions

The concept of local sleep was initially sparked by the finding that aquatic mammals sleep with one hemisphere at the time when in the water. This was followed by several different experiments exploring the idea. We are now in a situation where this is measurable from the single neuron up to the hemisphere level, where deviations from the behavior of the majority of neurons or differences between cortical regions in EEG activity or states are all called local sleep. It may, however, be that the dissident neurons that we observe in the global state are merely expressions of transitions between states that are in progress. In addition, there is probably a fundamental difference between local differences in EEG activity (i.e., slow waves during NREM sleep), but remaining in the same state and local differences in vigilance states (waking vs. NREM sleep) between cortical regions. These differences are not only on the functional level, but also on the mechanistic level.

When looking at the phenomenon of local sleep in this way, clearer defined questions can be asked when investigating the mechanisms behind the different types of local sleep. They should be concentrated on differences at the local cortical level, but also investigate the behavior of deeper brain areas related to ARAS and areas involved in the flip‐flop sleep mechanism.

**What is an electroencephalographic slow wave?**

The EEG is a representation of the activity of a large group of neurons underneath the recording electrode. This electrode can be glued on the scalp, or be placed through the scull on the dura. It is even possible to perform EEG type of recordings deeper in the brain. However, here we will limit ourselves to cortical activity. Only when the neurons under the electrode are neatly spaced in the same direction, as is the case in the cortex, and shows a certain level of synchrony, it is possible to decern waves within the recorded signal. The amplitude and frequency of these waves depend on the type of activity of the cortex, which usually correlates with the vigilance state of the brain. Part of sleep science is based on the fact that these wave patterns differ between sleep and waking and between NREM sleep and REM sleep.

Cortical neuronal firing patterns in NREM sleep show cortical networks alternating between periods of generalized population firing on top of depolarizations of the neuronal membranes, and periods of silence and hyperpolarization of the neuronal membrane (Steriade et al. 2001; Harris and Thiele 2011; Crunelli and Hughes 2010). This synchronized pattern of neuronal activity produces the typical EEG oscillations at frequencies below 5 Hz, which are generally called slow waves. Thes periods of enhanced activity are in the literature referred to as “on periods” and the inactive, or silent periods as “off periods” (Vyazovskiy and Harris 2013). On and off periods are not only associated with EEG slow waves, but also with depolarized and hyperpolarized membrane potentials of individual neurons, which are in their turn referred to as “up” and “down” states (Chauvette et al. 2010; Poulet and Petersen 2008; Saleem et al. 2010; Okun and Lampl 2008). In addition, EEG slow waves are not simple standing waves on a surface, but are travelling waves, mostly initiated at the frontal cortex and then spreading out and moving backward over the cortex (Massimini et al. 2004; Nir et al. 2011).

## Author Contributions

**Tom Deboer**: conceptualization, investigation, writing – original draft, visualization, writing – review and editing, supervision.

## Funding

The author has nothing to report.

## Conflicts of Interest

The author declares no conflicts of interest.

## Data Availability

Data sharing not applicable to this article as no datasets were generated or analysed during the current study.

## References

[brb371362-bib-0001] Aston‐Jones, G. , and F. E. Bloom . 1981. “Activity of Norepinephrine‐Containing Locus Coeruleus Neurons in Behaving Rats Anticipates Fluctuations in the Sleep‐waking Cycle.” Journal of Neuroscience 1: 876–886. 10.1523/JNEUROSCI.01-08-00876.1981. 7346592 PMC6564235

[brb371362-bib-0002] Benington, J. H. , and H. Craig Heller . 1995. “Restoration of Brain Energy Metabolism as the Function of Sleep.” Progress in Neurobiology 45: 347–360. 10.1016/0301-0082(94)00057-o. 7624482

[brb371362-bib-0003] Borbely, A. A. 1982. “A Two Process Model of Sleep Regulation.” Human Neurobiology 1: 195–204. 7185792

[brb371362-bib-0004] Borbély, A. A. , S. Daan , A. Wirz‐Justice , and T. Deboer . 2016. “The Two‐Process Model of Sleep Regulation: A Reappraisal.” Journal of Sleep Research 25: 131–143. 10.1111/jsr.12371. 26762182

[brb371362-bib-0005] Cajochen, C. , D. P. Brunner , K. Krauchi , P. Graw , and A. Wirz‐Justice . 1995. “Power Density in Theta/Alpha Frequencies of the Waking EEG Progressively Increases During Sustained Wakefulness.” Sleep 18: 890–894. 10.1093/sleep/18.10.890. 8746397

[brb371362-bib-0006] Cajochen, C. , J. K. Wyatt , C. A. Czeisler , and D. J. Dijk . 2002. “Separation of Circadian and Wake Duration‐Dependent Modulation of EEG Activation During Wakefulness.” Neuroscience 114: 1047–1060. 10.1016/s0306-4522(02)00209-9. 12379258

[brb371362-bib-0007] Chauvette, S. , M. Volgushev , and I. Timofeev . 2010. “Origin of Active States in Local Neocortical Networks During Slow Sleep Oscillation.” Cerebral Cortex 20: 2660–2674. 10.1093/cercor/bhq009. 20200108 PMC2951844

[brb371362-bib-0008] Crunelli, V. , and S. W. Hughes . 2010. “The Slow (<1 Hz) Rhythm of Non‐REM Sleep: A Dialogue Between Three Cardinal Oscillators.” Nature Neuroscience 13: 9–17. 10.1038/nn.2445. 19966841 PMC2980822

[brb371362-bib-0009] Deboer, T. 2020. “Circadian Regulation of Sleep in Mammals.” Current Opinion in Physiology 15: 89–95. 10.1016/j.cophys.2019.12.015.

[brb371362-bib-0010] Deboer, T. 2025. “Sleep Homeostatic and Circadian Clock Changes Can be Obtained by Manipulating One Single Kinase, but Do the Two Processes Meet Each Other There?” Sleep 48: zsae291. 10.1093/sleep/zsae291. 39673772 PMC11807888

[brb371362-bib-0011] Deboer, T. , S. Overeem , N. A. H. Visser , et al. 2004. “Convergence of Circadian and Sleep Regulatory Mechanisms on Hypocretin‐1.” Neuroscience 129: 727–732. 10.1016/j.neuroscience.2004.07.049. 15541893

[brb371362-bib-0012] Durán, E. , C. N. Oyanedel , N. Niethard , M. Inostroza , and J. Born . 2018. “Sleep Stage Dynamics in Neocortex and Hippocampus.” Sleep 41: zsy060. 10.1093/sleep/zsy060. 29893972

[brb371362-bib-0013] Emrick, J. J. , B. A. Gross , B. T. Riley , and G. R. Poe . 2016. “Different Simultaneous Sleep States in the Hippocampus and Neocortex.” Sleep 39: 2201–2209. 10.5665/sleep.6326. 27748240 PMC5103808

[brb371362-bib-0014] Funk, C. M. , S. Honjoh , A. V. Rodriguez , C. Cirelli , and G. Tononi . 2016. “Local Slow Waves in Superficial Layers of Primary Cortical Areas During REM Sleep.” Current Biology 26: 396–403. 10.1016/j.cub.2015.11.062. 26804554 PMC4747819

[brb371362-bib-0015] Gaus, S. E. , R. E. Strecker , B. A. Tate , R. A. Parker , and C. B. Saper . 2002. “Ventrolateral Preoptic Nucleus Contains Sleep‐Active, Galaninergic Neurons in Multiple Mammalian Species.” Neuroscience 115: 285–294. 10.1016/s0306-4522(02)00308-1. 12401341

[brb371362-bib-0016] Hallanger, A. E. , A. I. Levey , H. J. Lee , D. B. Rye , and B. H. Wainer . 1987. “The Origins of Cholinergic and Other Subcortical Afferents to the Thalamus in the Rat.” Journal of Comparative Neurology 262: 105–124. 10.1002/cne.902620109. 2442206

[brb371362-bib-0017] Harris, K. D. , and A. Thiele . 2011. “Cortical State and Attention.” Nature Reviews Neuroscience 12: 509–523. 10.1038/nrn3084. 21829219 PMC3324821

[brb371362-bib-0018] Huber, R. , T. Deboer , and I. Tobler . 2000. “Topography of EEG Dynamics After Sleep Deprivation in Mice.” Journal of Neurophysiology 84: 1888–1893. 10.1152/jn.2000.84.4.1888. 11024081

[brb371362-bib-0019] Huber, R. , M. Felice Ghilardi , M. Massimini , and G. Tononi . 2004. “Local Sleep and Learning.” Nature 430: 78–81. 10.1038/nature02663. 15184907

[brb371362-bib-0020] Huber, R. , M. F. Ghilardi , M. Massimini , et al. 2006. “Arm Immobilization Causes Cortical Plastic Changes and Locally Decreases Sleep Slow Wave Activity.” Nature Neuroscience 9: 1169–1176. 10.1038/nn1758. 16936722

[brb371362-bib-0021] Jones, B. E. 2003. “Arousal Systems.” Frontiers in Bioscience 8: 438–451. 10.2741/1074. 12700104

[brb371362-bib-0022] Kattler, H. , D.‐J. Dijk , and A. A. Borbély . 1994. “Effect of Unilateral Somatosensory Stimulation Prior to Sleep on the Sleep EEG in Humans.” Journal of Sleep Research 3: 159–164. 10.1111/j.1365-2869.1994.tb00123.x. 10607121

[brb371362-bib-0023] Krueger, J. M. , F. Obál , and J. Fang . 1999. “Why We Sleep: A Theoretical View of Sleep Function.” Sleep Medicine Reviews 3: 119–129. 10.1016/s1087-0792(99)90019-9. 15310481

[brb371362-bib-0024] Lapierre, J. L. , P. O. Kosenko , O. I. Lyamin , T. Kodama , L. M. Mukhametov , and J. M. Siegel . 2007. “Cortical Acetylcholine Release Is Lateralized During Asymmetrical Slow‐Wave Sleep in Northern Fur Seals.” Journal of Neuroscience 27: 11999–12006. 10.1523/JNEUROSCI.2968-07.2007. 17978041 PMC6673386

[brb371362-bib-0025] Luppi, P.‐H. , O. Clément , E. Sapin , et al. 2011. “The Neuronal Network Responsible for Paradoxical Sleep and Its Dysfunctions Causing Narcolepsy and Rapid Eye Movement (REM) Behavior Disorder.” Sleep Medicine Reviews 15: 153–163. 10.1016/j.smrv.2010.08.002. 21115377

[brb371362-bib-0026] Lyamin, O. I. , J. L. Lapierre , P. O. Kosenko , et al. 2016. “Monoamine Release During Unihemispheric Sleep and Unihemispheric Waking in the Fur Seal.” Sleep 39: 625–636. 10.5665/sleep.5540. 26715233 PMC4763370

[brb371362-bib-0027] Lyamin, O. I. , J. L. Lapierre , P. O. Kosenko , L. M. Mukhametov , and J. M. Siegel . 2008. “Electroencephalogram Asymmetry and Spectral Power During Sleep in the Northern Fur Seal.” Journal of Sleep Research 17: 154–165. 10.1111/j.1365-2869.2008.00639.x. 18482104

[brb371362-bib-0028] Massimini, M. , R. Huber , F. Ferrarelli , S. Hill , and G. Tononi . 2004. “The Sleep Slow Oscillation as a Traveling Wave.” Journal of Neuroscience 24: 6862–6870. 10.1523/JNEUROSCI.1318-04.2004. 15295020 PMC6729597

[brb371362-bib-0029] McCormick, D. A. 1989. “Cholinergic and Noradrenergic Modulation of Thalamocortical Processing.” Trends in Neuroscience 12: 215–221. 10.1016/0166-2236(89)90125-2. 2473557

[brb371362-bib-0030] Mukhametov, L. M. , A. Y. Supin , and I. G. Polyakova . 1977. “Interhemispheric Asymmetry of the Electroencephalographic Sleep Patterns in Dolphins.” Brain Research 134: 581–584. 10.1016/0006-8993(77)90835-6. 902119

[brb371362-bib-0031] Nir, Y. , R. J. Staba , T. Andrillon , et al. 2011. “Regional Slow Waves and Spindles in Human Sleep.” Neuron 70: 153–169. 10.1016/j.neuron.2011.02.043. 21482364 PMC3108825

[brb371362-bib-0032] Nobili, L. , L. De Gennaro , P. Proserpio , et al. 2012. “Local Aspects of Sleep: Observations From Intracerebral Recordings in Humans.” Progress in Brain Research 199: 219–232. 10.1016/B978-0-444-59427-3.00013-7. 22877668

[brb371362-bib-0033] Nobili, L. , M. Ferrara , F. Moroni , et al. 2011. “Dissociated Wake‐Like and Sleep‐Like Electro‐Cortical Activity During Sleep.” Neuroimage 58: 612–619. 10.1016/j.neuroimage.2011.06.032. 21718789

[brb371362-bib-0034] Oikonomou, G. , M. Altermatt , R. W. Zhang , et al. 2019. “The Serotonergic Raphe Promote Sleep in Zebrafish and Mice.” Neuron 103: 686–701.e8. 10.1016/j.neuron.2019.05.038. 31248729 PMC6706304

[brb371362-bib-0035] Okun, M. , and I. Lampl . 2008. “Instantaneous Correlation of Excitation and Inhibition During Ongoing and Sensory‐Evoked Activities.” Nature Neuroscience 11: 535–537. 10.1038/nn.2105. 18376400

[brb371362-bib-0036] Oleksenko, A. I. , L. M. Mukhametov , I. G. Polyakova , A. Y. Supin , and V. M. Kovalzon . 1992. “Unihemispheric Sleep Deprivation in Bottlenose Dolphins.” Journal of Sleep Research 1: 40–44. 10.1111/j.1365-2869.1992.tb00007.x. 10607024

[brb371362-bib-0037] Palchykova, S. , T. Deboer , and I. Tobler . 2002. “Selective Sleep Deprivation After Daily Torpor in the Djungarian Hamster.” Journal of Sleep Research 11: 313–319. 10.1046/j.1365-2869.2002.00310.x. 12464099

[brb371362-bib-0038] Pigarev, I. N. , H. C. Nothdurft , and S. Kastner . 1997. “Evidence for Asynchronous Development of Sleep in Cortical Areas.” Neuroreport 8: 2557–2560. 10.1097/00001756-199707280-00027. 9261826

[brb371362-bib-0039] Poulet, J. F. , and C. C. Petersen . 2008. “Internal Brain State Regulates Membrane Potential Synchrony in Barrel Cortex of Behaving Mice.” Nature 454: 881–885. 10.1038/nature07150. 18633351

[brb371362-bib-0040] Reichert, C. F. , T. Deboer , and H. P. Landolt . 2022. “Adenosine, Caffeine, and Sleep‐Wake Regulation: State of the Science and Perspectives.” Journal of Sleep Research 31: e13597. 10.1111/jsr.13597. 35575450 PMC9541543

[brb371362-bib-0041] Rusterholz, T. , and P. Achermann . 2011. “Topographical Aspects in the Dynamics of Sleep Homeostasis in Young Men: Individual Patterns.” BMC Neuroscience 12: 84. 10.1186/1471-2202-12-84. 21846365 PMC3173373

[brb371362-bib-0042] Saleem, A. B. , P. Chadderton , J. Apergis‐Schoute , K. D. Harris , and S. R. Schultz . 2010. “Methods for Predicting Cortical UP and DOWN States From the Phase of Deep Layer Local Field Potentials.” Journal of Computational Neuroscience 29: 49–62. 10.1007/s10827-010-0228-5. 20225075 PMC3094772

[brb371362-bib-0043] Saper, C. B. , T. E. Scammell , and J. Lu . 2005. “Hypothalamic Regulation of Sleep and Circadian Rhythms.” Nature 437: 1257–1263. 10.1038/nature04284. 16251950

[brb371362-bib-0044] Serantes, D. , M. Cavelli , J. Gonzalez , A. Mondino , L. Benedetto , and P. Torterolo . 2025. “Characterising the Power Spectrum Dynamics of the Non‐REM to REM Sleep Transition.” Journal of Sleep Research 34: e14388. 10.1111/jsr.14388. 39520222

[brb371362-bib-0045] Sherin, J. E. , J. K. Elmquist , F. Torrealba , and C. B. Saper . 1998. “Innervation of Histaminergic Tuberomammillary Neurons by GABAergic and Galaninergic Neurons in the Ventrolateral Preoptic Nucleus of the Rat.” Journal of Neuroscience 18: 4705–4721. 10.1523/JNEUROSCI.18-12-04705.1998. 9614245 PMC6792696

[brb371362-bib-0046] Steininger, T. L. , M. N. Alam , H. Gong , R. Szymusiak , and D. McGinty . 1999. “Sleep‐Waking Discharge of Neurons in the Posterior Lateral Hypothalamus of the Albino Rat.” Brain Research 840: 138–147. 10.1016/s0006-8993(99)01648-0. 10517961

[brb371362-bib-0047] Steriade, M. , I. Timofeev , and F. Grenier . 2001. “Natural Waking and Sleep States: A View From Inside Neocortical Neurons.” Journal of Neurophysiology 85: 1969–1985. 10.1152/jn.2001.85.5.1969. 11353014

[brb371362-bib-0048] Strijkstra, A. M. , D. G. Beersma , B. Drayer , N. Halbesma , and S. Daan . 2003. “Subjective Sleepiness Correlates Negatively With Global Alpha (8–12 Hz) and Positively With Central Frontal Theta (4–8 Hz) Frequencies in the Human Resting Awake Electroencephalogram.” Neuroscience Letters 340: 17–20. 10.1016/s0304-3940(03)00033-8. 12648748

[brb371362-bib-0049] Szymusiak, R. , N. Alam , T. L. Steininger , and D. McGinty . 1998. “Sleep‐Waking Discharge Patterns of Ventrolateral Preoptic/Anterior Hypothalamic Neurons in Rats.” Brain Research 803: 178–188. 10.1016/s0006-8993(98)00631-3. 9729371

[brb371362-bib-0050] Tamaki, M. , J. W. Bang , T. Watanabe , and Y. Sasaki . 2016. “Night Watch in One Brain Hemisphere During Sleep Associated With the First‐Night Effect in Humans.” Current Biology 26: 1190–1194. 10.1016/j.cub.2016.02.063. 27112296 PMC4864126

[brb371362-bib-0051] Tononi, G. , and C. Cirelli . 2006. “Sleep Function and Synaptic Homeostasis.” Sleep Medicine Reviews 10: 49–62. 10.1016/j.smrv.2005.05.002. 16376591

[brb371362-bib-0052] Tzioridou, S. , T. Campillo‐Ferrer , J. Cañas‐Martín , et al. 2025. “The Clinical Neuroscience of Lucid Dreaming.” Neuroscience and Biobehavioral Reviews 169: 106011. 10.1016/j.neubiorev.2025.106011. 39818345

[brb371362-bib-0053] Vyazovskiy, V. V. , and K. D. Harris . 2013. “Sleep and the Single Neuron: The Role of Global Slow Oscillations in Individual Cell Rest.” Nature Reviews Neuroscience 14: 443–451. 10.1038/nrn3494. 23635871 PMC3972489

[brb371362-bib-0054] Vyazovskiy, V. V. , U. Olcese , E. C. Hanlon , Y. Nir , C. Cirelli , and G. Tononi . 2011. “Local Sleep in Awake Rats.” Nature 472: 443–447. 10.1038/nature10009. 21525926 PMC3085007

[brb371362-bib-0055] Vyazovskiy, V. V. , G. Ruijgrok , T. Deboer , and I. Tobler . 2006. “Running Wheel Accessibility Affects the Regional Electroencephalogram During Sleep in Mice.” Cerebral Cortex 16: 328–336. 10.1093/cercor/bhi110. 15901653

[brb371362-bib-0056] Vyazovskiy, V. V. , and I. Tobler . 2008. “Handedness Leads to Interhemispheric EEG Asymmetry During Sleep in the Rat.” Journal of Neurophysiology 99: 969–975. 10.1152/jn.01154.2007. 18077659

[brb371362-bib-0057] Vyazovskiy, V. V. , E. Welker , J. M. Fritschy , and I. Tobler . 2004. “Regional Pattern of Metabolic Activation Is Reflected in the Sleep EEG After Sleep Deprivation Combined With Unilateral Whisker Stimulation in Mice.” European Journal of Neuroscience 20: 1363–1370. 10.1111/j.1460-9568.2004.03583.x. 15341608

[brb371362-bib-0058] Zeitzer, J. M. , C. L. Buckmaster , K. J. Parker , C. M. Hauck , D. M. Lyons , and E. Mignot . 2003. “Circadian and Homeostatic Regulation of Hypocretin in a Primate Model: Implications for the Consolidation of Wakefulness.” Journal of Neuroscience 23: 3555–3560. 10.1523/JNEUROSCI.23-08-03555.2003. 12716965 PMC6742340

